# Cervical necrotising fasciitis and descending mediastinitis secondary to unilateral tonsillitis: a case report

**DOI:** 10.1186/1752-1947-2-368

**Published:** 2008-12-04

**Authors:** Asad Islam, Michael Oko

**Affiliations:** 1 Pilgrim hospital, Sibsey road, Boston, Lincolnshire, PE21 9QS, UK

## Abstract

**Introduction:**

Cervical necrotizing fasciitis is an aggressive infection with high morbidity and mortality. We present a case of cervical necrotizing fasciitis and descending mediastinitis in a healthy young man, caused by unilateral tonsillitis with a successful outcome without aggressive debridement.

**Case presentation:**

A 41-year-old man was admitted to our unit with a diagnosis of severe acute unilateral tonsillitis. On admission, he had painful neck movements and the skin over his neck was red, hot and tender. Computed tomography scan of his neck and chest showed evidence of cervical necrotizing fasciitis and descending mediastinitis secondary to underlying pharyngeal disease. He was treated with broad-spectrum intravenous antibiotics. His condition improved over the next 3 days but a tender and fluctuant swelling appeared in the suprasternal region. A repeat scan showed the appearance of an abscess extending from the pretracheal region to the upper mediastinum which was drained through a small transverse anterior neck incision. After surgery, the patient's condition quickly improved and he was discharged on the 18th day of admission.

**Conclusion:**

Less invasive surgical techniques may replace conventional aggressive debridement as the treatment of choice for cervical necrotizing fasciitis and descending necrotizing mediastinitis.

## Introduction

Cervical necrotizing fasciitis (CNF) is an uncommon but aggressive infection with high morbidity and mortality. We present a case of CNF and descending mediastinitis in a healthy young patient, caused by unilateral tonsillitis, with a successful outcome involving simple incision and drainage. We discuss the importance of a computerised tomography (CT) scan in making an early diagnosis. We also review the possible role of less aggressive surgical techniques in the management of CNF.

## Case presentation

A 41-year-old man was admitted with a 3-day history of severe sore throat and painful swallowing. According to him, it started as a mild sore throat but rapidly worsened despite taking oral penicillin for 3 days from his general practitioner (GP). There was no significant past medical history. On examination, his temperature was 38.3°C and he was tachycardic (102/min). His neck movements were markedly restricted by pain and he had a spiking fever. The skin on the front of his neck was red, hot and tender down to his clavicles, with no evidence of localised swelling or ischaemia/necrosis. Oral examination showed a markedly inflamed right tonsil covered with patchy grey exudate. There was no clinical evidence of peritonsillar abscess. The left tonsil appeared remarkably normal. Flexible nasendoscopy showed normal epiglottis and larynx.

Blood tests revealed leukocytosis of 22.3 × 10^9 ^cells/L and a high C-reactive protein (CRP) of 421 mg/L.

**Figure 1:**
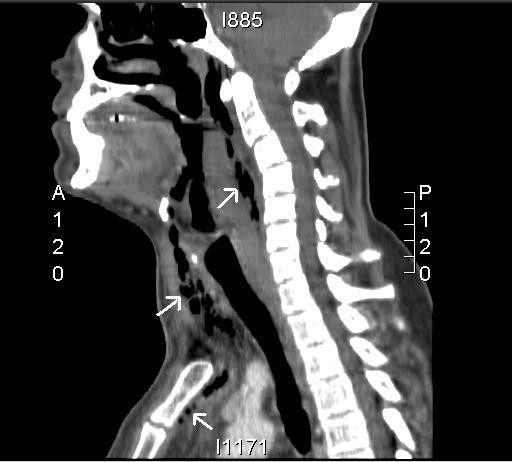
Sagittal reconstruction of CT of the neck and upper mediastinum; extensive soft tissue abnormality (swelling and surgical emphysema) in the retropharynx with loss of normal fat plane with the prevertebral space and possible communication with the oropharynx. Air (arrows) is also present in the pretracheal tissue and extends into the upper mediastinum.

**Figure 2:**
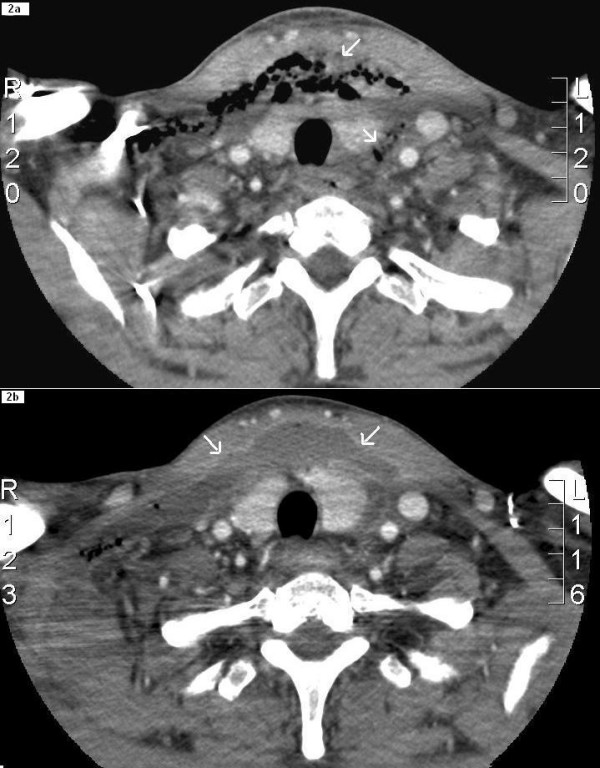
2a: Horizontal CT view of the neck at the level of suprasternal notch; the surgical emphysema and soft tissue swelling (arrows) is seen in the pretracheal region and prevertebral space and is extending beyond the carotid sheaths. In 2b (after 3 days of antibiotic therapy), the appearance of the disease process has changed (in comparison to 2a) with a decreasing amount of air in the soft tissues and replacement by an abscess (arrows) which has irregular enhancing margins.

**Figure 3:**
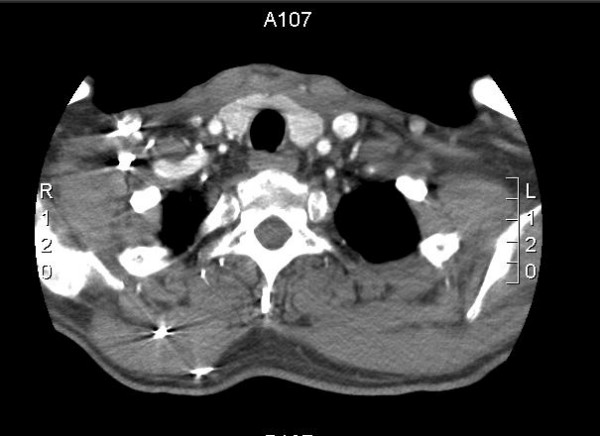
Horizontal CT view of the neck at the level of suprasternal notch (about 6 weeks after surgery). This shows complete resolution of the inflammatory process.

A lateral X-ray of his neck showed air and swelling in the pretracheal soft tissues and loss of normal cervical lordosis. These findings prompted an urgent CT scan of his neck and chest, which demonstrated air shadows and diffuse swelling and enhancement of cervical fascia extending from skull base to upper mediastinum. Similar changes were also seen in the pretracheal soft tissue (figures 1, 2 and 3).

Throat swabs and blood cultures were sent for microbiology and intravenous (I.V.) therapy with broad spectrum antibiotics was commenced as it was decided to initially treat the condition medically. Surgical debridement was not carried out at this stage because there was no skin necrosis and no collection was shown by the CT scan.

His general condition showed improvement. His temperature came down and his oral intake improved but over the next 3 days a tender and fluctuant swelling appeared in the suprasternal region with diffuse margins.

A repeat CT scan of the neck and chest at this stage showed receding air shadows and the appearance of an abscess extending from the pretracheal region to the upper mediastinum. After discussions with a radiologist it was decided to carry out a limited surgery in the form of incision and drainage. About 200 ml of pus was drained via a small transverse suprasternal incision and midline split of the abscess wall. All the loculi were broken down with a finger. Cultures of the pus did not reveal any organisms.

After surgery, the patient's condition dramatically improved. Drains from the wound were removed on the fifth postoperative day and the wound was allowed to heal by secondary intention. Inflammatory markers progressively came back to normal and the patient was discharged on the 18th day after admission. He was followed up after 2 weeks with a repeat CT scan which showed complete resolution. He underwent "interval tonsillectomy" in our department and the tonsils were sent for histopathology which did not reveal any abnormal findings.

## Discussion

Necrotizing fasciitis (NF) is a fulminant infection that affects the deep and superficial fascia while initially sparing the overlying skin and underlying muscle. The most common sites for NF to occur are the abdomen, extremities and perineum. It is uncommon in the cervicofacial area and the usual nidus of infection in these cases is the teeth. The presence of immuno-compromising conditions predisposes to CNF as well as increases morbidity and mortality [1]. Peritonsillar abscess is a rare but recognized cause of this condition [2,3]. However, tonsillitis in our patient was not complicated by a peritonsillar abscess. Moreover, the tonsillitis was unilateral in our patient and none of the known causes of unilateral tonsillitis were found in him.

Soft tissue X-ray of the neck is a useful initial investigation in less suspicious cases and it can detect air in the soft tissues [3]. However, in suspicious cases, one should have a low threshold for performing a CT scan. The role of the CT scan in CNF is twofold, namely, to help establish the diagnosis at an early clinical stage and to help detect complications due to progressive tissue necrosis after initial surgical management. The most common CT findings in CNF are the thickening and infiltration of subcutaneous tissues, fluid collection in multiple neck compartments and diffuse enhancement and thickening of the cervical fascia, platysma and sternocleidomastoid and strap muscles. Inconsistent features include gas collection in the soft tissues [4,5]. In this case, none of the specimens grew any organisms. This was probably because the patient had started using antibiotics 3 days before admission. However, empirical antibiotics were chosen on the basis of well known synergism between aerobes and anaerobes [1,6,7]. Historically, early and sometimes multiple, radical debridement has remained the cornerstone in the management of this condition [1,3,8]. But two large studies done at Osaka University medical school in Japan recently reported that percutaneous catheter drainage as a novel treatment for CNF and descending necrotizing mediastinitis is less invasive than conventional surgical drainage and produced a similar outcome. Moreover, percutaneous catheter drainage areas are less likely to become secondarily infected by antibiotic-resistant bacteria, and it seems superior to surgical drainage in pain control and in preventing protein leakage from the wound [9,10]. Although the treatment strategy in our case was not exactly the same, we successfully avoided aggressive debridement. We suggest that there is certainly a place for less invasive surgical management in the treatment of CNF and mediastinitis but more research is needed to fully evaluate the effectiveness and suitability of less invasive treatment strategies.

## Conclusion

CNF is being increasingly reported from across the world. One should have a low threshold for CT scan in suspicious cases due to its easy and quick availability in most centres. Minimally invasive surgical techniques like percutaneous catheter drainage may replace conventional surgical drainage as the treatment of choice for CNF and descending necrotizing mediastinitis. More studies are needed to evaluate these novel treatments.

## Abbreviations

CT: computerised tomography; CRP: C-reactive protein; CNF: cervical necrotising fasciitis; GP: General Practitioner; I.V.: intravenous

## Consent

Written informed consent was obtained from the patient for publication of this case report and any accompanying images. A copy of the written consent is available for review by the Editor-in-Chief of this journal.

## Competing interests

The authors declare that they have no competing interests.

## Authors' contributions

AI wrote the abstract, case summary, literature review and discussion for this case report and arranged CT pictures. MO was the clinician responsible for the overall care of the patient and helped to draft the manuscript. Both authors read and approved the final manuscript.
